# Bidirectional Neuronal Control of Epileptiform Activity by Repetitive Transcranial Focused Ultrasound Stimulations

**DOI:** 10.1002/advs.202302404

**Published:** 2023-11-23

**Authors:** Taewon Choi, Minseok Koo, Jaesoon Joo, Taekyung Kim, Young‐Min Shon, Jinhyoung Park

**Affiliations:** ^1^ Department of Intelligent Precision Healthcare Convergence Sungkyunkwan University Suwon 16419 South Korea; ^2^ Department of Health Sciences and Technology Samsung Advanced Institute for Health Science and Technology Sungkyunkwan University Seoul 06351 South Korea; ^3^ Department of Neurology Samsung Medical Center Sungkyunkwan University School of Medicine Seoul 06351 South Korea; ^4^ Biomedical Engineering Research Center Samsung Medical Center Seoul 06351 South Korea; ^5^ Department of Medical Device Management and Research Samsung Advanced Institute for Health Science and Technology Sungkyunkwan University Seoul 06351 South Korea; ^6^ Department of Biomedical Engineering Sungkyunkwan University Suwon 16419 South Korea

**Keywords:** antiepileptic treatment, bidirectional neuronal control, epileptiform activity, transcranial focused ultrasound

## Abstract

Repetitive stimulation procedures are used in neuromodulation techniques to induce persistent excitatory or inhibitory brain activity. The directivity of modulation is empirically regulated by modifying the stimulation length, interval, and strength. However, bidirectional neuronal modulations using ultrasound stimulations are rarely reported. This study presents bidirectional control of epileptiform activities with repetitive transcranial‐focused ultrasound stimulations in a rat model of drug‐induced acute epilepsy. It is found that repeated transmission of elongated (40 s), ultra‐low pressure (0.25 MPa) ultrasound can fully suppress epileptic activities in electro‐encephalography and cerebral blood volume measurements, while the change in bursting intervals from 40 to 20 s worsens epileptic activities even with the same burst length. Furthermore, the suppression induced by 40 s long bursts is transformed to excitatory states by a subsequent transmission. Bidirectional modulation of epileptic seizures with repeated ultrasound stimulation is achieved by regulating the changes in glutamate and γ‐Aminobutyric acid levels, as confirmed by measurements of expressed c‐Fos and GAD65 and multitemporal analysis of neurotransmitters in the interstitial fluid obtained via microdialysis.

## Graphical abstract

Bidirectional control of epileptiform activities can be achieved by changing stimulus parameters for repetitive transcranial focused ultrasound. The repeated sonication of elongated burst with longer intervals can increase extracellular GABA and induce suppressive effects while the same burst with shorter intervals returns opposite responses. Furthermore, the suppressed epileptiform transforms the excitatory state by following stronger short bursts.

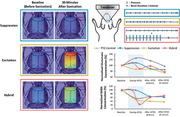

## 1 Introduction

Epilepsy, a common neurological disorder,^[^ ^1^ ^]^ is caused by the failure of neuronal inhibition, which disperses epileptic responses over broad brain regions.^[^ ^2^ ^]^ The primary features of antiepileptic therapies, such as antiepileptic drugs (AEDs),^[^ ^3^, ^4^ ^]^ vagus nerve stimulation (VNS),^[^ ^5^, ^6^ ^]^ deep brain stimulation (DBS),^[^ ^5^, ^7^ ^]^ transcranial magnetic stimulation (TMS), and transcranial direct/alternating current stimulation (tD/ACS),^[^ ^5^, ^8^, ^9^, ^10^ ^]^ involve alleviation of cortical hyper‐excitabilities by either enhancing γ‐Aminobutyric acid (GABA)ergic neurons or blocking excitatory amino acid neurotransmission. Optimal stimulation sequences for these conventional therapeutic approaches have been empirically investigated because their performance in seizure suppression relies on stimulation parameters, such as stimulus strength, length, and intervals. In addition, certain combinations of these parameters in AEDs,^[^ ^11^, ^12^ ^]^ VNS,^[^ ^13^ ^]^ DBS,^[^ ^14^ ^]^ and TMS^[^ ^15^, ^16^, ^17^ ^]^ can exacerbate epileptic symptoms. Meanwhile, transcranial‐focused ultrasound (tFUS) is a recent therapeutic approach that has gained significant attention for its antiepileptic effects in drug‐induced small animal epilepsy models,^[^ ^18^, ^19^, ^20^, ^21^, ^22^, ^23^, ^24^, ^25^ ^]^ and there are expectations that tFUS may be more efficient in antiepileptic therapeutic approach owing to its ability to stimulate deep brain structures within millimeter‐scale lesions. Despite the successful induction of suppressive effects and several efforts to optimize the stimulation parameters for tFUS, its efficacy in prior studies remains vague. In addition, some studies have indicated that the reliability of the neuromodulation effects may not be sufficient for clinical use because the sonication time for tFUS is too short to cause persistent modulation effects.^[^ ^21^, ^23^, ^24^ ^]^ Furthermore, similar to other stimulation approaches, bidirectional neuromodulation can be induced by tFUS. Care should be taken to avoid side effects because ultrasound stimulation also works on inhibitory pathways by activating GABAergic neurons or blocking ion channels, like other conventional stimulant methods,^[^ ^21^, ^22^, ^23^, ^24^ ^]^ and may exacerbate epileptic symptoms.

In the present study, repetitive tFUS (rtFUS) having the longer repetition ratio of tens of seconds had been explored to empirically demonstrate significant suppressive effects on epileptiform activities in a pentylenetetrazole (PTZ)‐induced acute generalized epilepsy model.^[^ ^26^ ^]^ As repetitive TMS (rTMS) exerts longer neuromodulatory effects than single‐pulse TMS in the treatment of major depressive disorders,^[^ ^27^, ^28^, ^29^ ^]^ similar repetitive stimulus sequences in ultrasound brain stimulation have recently been reported to have long‐lasting inhibitory effects on the anti‐saccade motion of non‐human primates.^[^ ^29^, ^30^ ^]^ Instead of a single stimulation burst, burst trains can be more effective in suppressing epileptiform activities, eventually resulting in symptoms that are comparable to those in the baseline. Therefore, the prolonged sustainability of the antiepileptic effects of rtFUS can be compared with that of single‐burst tFUS. Furthermore, paradoxical proconvulsive effects and even a transition from anticonvulsive to proconvulsive effects may be discovered by navigating changes in rtFUS parameters, such as the interval between stimulus bursts, burst duration, and strength because these parameters are known to affect GABA secretion and induce bidirectional epileptiform activities in other stimulation methods (such as tDCS and TMS^[^ ^2^, ^14^, ^31^, ^32^, ^33^, ^34^ ^]^). The exploratory studies for comparison in epileptic activities between different acoustic parameters of rtFUS (e.g., stronger vs weaker bursts, longer vs shorter burst length, or burst interval) will help in identifying the optimal transmission conditions to perform antiepileptic effects.

Furthermore, we assessed the sustainability and bidirectional modulations in epileptic activities with rtFUS using electroencephalography (EEG) and optical measurements of cerebral blood volume (CBV) changes in the prefrontal cortex, where epileptiform activities in the hippocampus are reflected through the limbic system.^[^ ^35^ ^]^ Immunohistochemistry (IHC) was performed to visualize the excitatory and inhibitory activation of neurons in the hippocampus. In addition, changes in the amounts of excitatory (glutamate) and inhibitory (GABA) neurotransmitters during bidirectional modulation of epileptiform activities were assessed through multitemporal harvesting of interstitial fluid (ISF) from stimulated locations via microdialysis.

## 2 Results

**Figure 1:**
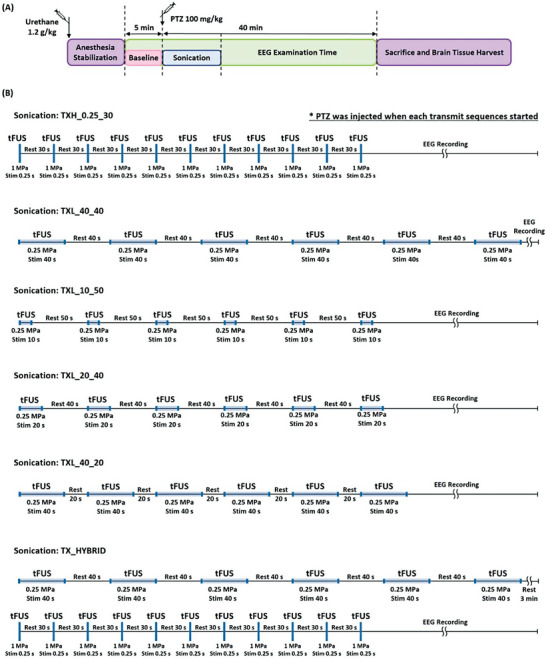
Sonication and EEG recording protocol. A) Summary of the overall experimental protocols for sonication and EEG recording. B) Detailed timeline of rtFUS stimulation and EEG recording for all transmit condition groups. TXH_0.25_30 is a sequence repeating eleven times of 1 MPa, 0.25 s bursts with 30 s intervals. TXL_40_40 is a sequence repeating six times of 0.25 MPa, 40 s bursts with 40 s intervals. TXL_10_50 is a sequence repeating six times of 0.25 MPa, 10 s bursts with 50 s intervals. TXL_20_40 is a sequence repeating six times of 0.25 MPa, 20 s bursts with 40 s intervals. TXL_40_20 is a sequence repeating six times of 0.25 MPa, 40 s bursts with 20 s intervals. TX_HYBRID is a sequence that consecutively applies TXL_40_40 and TXH_0.25_30 with 3 min resting period in between the two sequences.

Ultrasound stimulation was applied using a custom‐made focused ultrasound transducer over the right hemisphere of the midbrain to target the anterior thalamic nucleus (ATN). EEG was performed before, during, and after sonication. IHC was performed on brain samples harvested from the model animals. Optical measurements of CBV changes and microdialysis for proteomic analysis of neurotransmitters were performed in separate trials to measure the responses to the stimulation. The detailed repetitive transcranial ultrasound stimulation transmission sequences used in the experiment are shown in **Figure** **1**. The detailed experimental configurations are described in the Experimental Section and Supporting Information figures (Figures S1 and S2, Supporting Information).

### 2.1 Repeated Transcranial Low‐Intensity Elongated Ultrasound Stimulations for Sustainable Full Suppression of Epileptiform Activities

**Figure 2:**
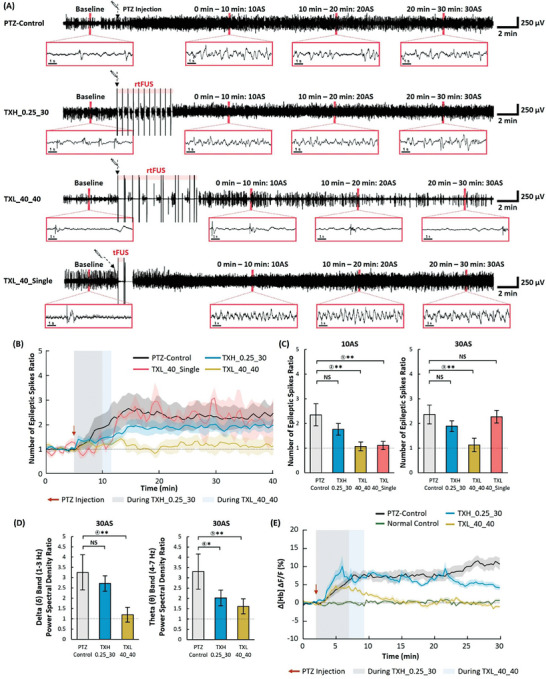
Changes in epileptiform activities during and after TXH_0.25_30, TXL_40_40, and TXL_40_Single. A) Time courses of EEG signals in the PTZ‐control and stimulated experimental groups. B) Time courses of the number of epileptic spikes ratio in each group (PTZ‐control: *n* = 8, TX groups: *n* = 5). C) Ratio of the number of epileptic spikes measured at time periods of 0–10 min (10AS) and 10–20 min (20AS) (PTZ‐control: *n* = 8, TX groups: *n* = 5). D) Power spectral density ratio of EEG signals at the delta (0.5–3 Hz) and theta (4–7 Hz) frequency bands (PTZ‐control: *n* = 8, TX groups: *n* = 5). E) Changes in the cerebral blood volume [Hb] calculated from 530 nm reflectance data (PTZ‐control: *n* = 6, TX groups: *n* = 6). Data presented as mean ± SEM, *p*‐values calculated via two‐tailed Mann–Whitney U test, ^**^ : *p* <0.05, * : *p* <0.10, NS : *p*> 0.10. *p* = ① 0.036 ② 0.030 ③ 0.030 ④ 0.045 ⑤ 0.019 ⑥ 0.093.

Changes in epileptiform activities were compared between the two stimulant sequences. The first sequence (TXH_0.25_30) comprised eleven repetitions of a stronger (1 MPa) and shorter (0.25 s) burst with a burst interval of 30 s. The second sequence (TXL_40_40) consisted of six repetitions of weaker (0.25 MPa) but longer (40 s) burst with a burst interval of 40 s (Figure 1). First, epileptiform activities were reliably induced in a PTZ‐control group that received PTZ, a drug used for acute seizure induction, without ultrasound stimulation. While sparsely located discharge peaks in the EEG signal were identified during the baseline period, the signal was transformed into ictal shapes that demonstrated repetitive high‐frequency discharge peaks in the PTZ‐control group (**Figure** **2**A). The relative number of epileptic spikes increased 2.6‐fold in the first 10 min after PTZ injection in the PTZ‐control group. The number then decreased for 7 min and saturated at 2.3 times the baseline (Figure 2B). Although the number decreased slightly from 10 to 17 min after the PTZ injection, this decrease was not statistically significant at 17 min after the PTZ injection compared to 10 min after the PTZ injection (*p* = 0.588; two‐tailed paired *t*‐test) where the number of epileptic spikes ratio still remained significantly different at 17 min after the PTZ injection compared to the baseline period (*p* = 0.014; two‐tailed paired *t*‐test). Following stimulation with TXH_0.25_30, the amplitude of the discharge pattern slightly reduced but gradually became comparable to that of the PTZ‐control group. During the period between 0 and 10 min post‐stimulation (10AS) and 20 and 30 min post‐stimulation (30AS), the number of epileptic spikes ratio in the TXH_0.25_30 group was not significantly different from that of the PTZ‐control (*p* = 0.524 and 0.724 at 10AS and 30AS, respectively; two‐tailed Mann–Whitney U test) (Figure 2C). Meanwhile, the discharging patterns following TXL_40_40 became similar to those of the baseline, and the suppressive effect lasted until the end of the EEG recording. TXL_40_40 administration resulted in the maintenance of the number of epileptic spikes ratio similar to that of the baseline, and a suppressive effect was still found at 10AS and 30AS (*p* = 0.030 and 0.030 at 10AS and 30AS compared with that of PTZ‐control, respectively; two‐tailed Mann–Whitney U test). In the delta band between 1 and 3 Hz, the spectral power with TXH_0.25_30 at 30AS was not significantly different from that of the PTZ control (*p* = 0.833; two‐tailed Mann‐Whitney U test). However, the relative spectral power followed by TXL_40_40 was 1.19 at 30AS, indicating that this level was close to the baseline. TXL_40_40 also demonstrated strong suppressive effects on the theta band between 4 and 7 Hz (*p* = 0.019 at 30AS compared with that of PTZ‐control; two‐tailed Mann–Whitney U test), whereas TXH_0.25_30 demonstrated mild effects (*p* = 0.093 at 30AS compared with that of PTZ‐control; two‐tailed Mann–Whitney U test) (Figure 2D). However, with a single transmission of an elongated (40 s), low pressure (0.25 MPa) burst, the number of epileptic spikes ratio became similar to that of the PTZ‐control group, although the suppressive effects were briefly observed for 5 min immediately after the stimulation (Figure 2B,C). In the CBV measurements, while the total hemoglobin amount (Δ[Hb]) barely changed in the normal control group (that did not receive PTZ or ultrasound stimulations), Δ[Hb] sharply increased in the PTZ‐control group after drug administration. The increase in Δ[Hb] was as low as that in the normal control group when TXL_40_40 was used (*p* = 0.699 at 20AS compared with that of normal control; two‐tailed Mann–Whitney U‐test), while the low Δ[Hb] level was maintained until the end of the recording. Following TXH_0.25_30, the Δ[Hb] level was similar to that of the PTZ control (*p* = 0.180 at 20AS; two‐tailed Mann–Whitney U test), although a gradual decay pattern was observed after the sonication was completed (Figure 2E).

### 2.2 Efficacy of rtFUS for Epilepsy Treatment is Dependent on the Burst Length in the rtFUS Sequence

**Figure 3:**
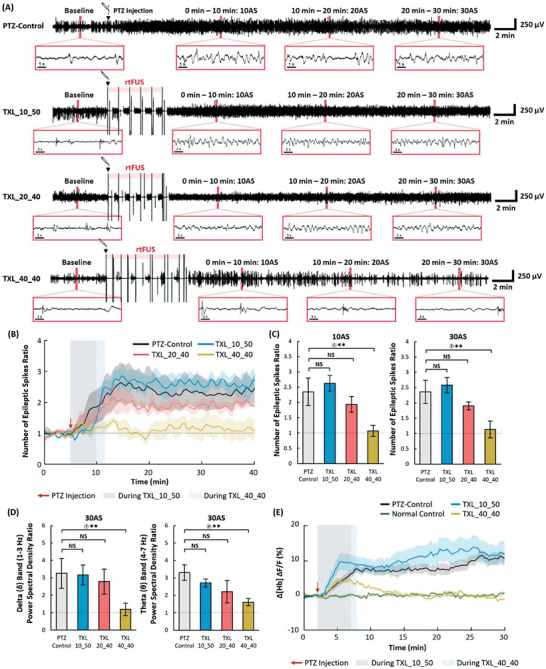
Changes in epileptiform activities during and after TXL_10_50, TXL_20_40, and TXL_40_40. A) Time courses of EEG signals in the PTZ‐control and stimulated experimental groups. B) Time courses of the number of epileptic spikes ratio in each group (PTZ‐control: *n* = 8, TX groups: *n* = 5). C) Ratio of the number of epileptic spikes measured at time periods of 0–10 min (10AS) and 10–20 min (20AS) (PTZ‐control: *n* = 8, TX groups: *n* = 5). D) Power spectral density ratio of EEG signals at the delta (0.5–3 Hz) and theta (4–7 Hz) frequency bands (PTZ‐control: *n* = 8, TX groups: *n* = 5). E) Changes in the cerebral blood volume [Hb] calculated from 530 nm reflectance data (PTZ‐control: *n* = 6, TX groups: *n* = 6). Data presented as mean ± SEM, *p*‐values calculated via two‐tailed Mann‐Whitney U test, ** : *p* <0.05, * : *p* <0.10, NS : *p*> 0.10. *p* = ① 0.030 ② 0.030 ③ 0.045 ④ 0.019.

To investigate the effects of burst length changes in rtFUS, the modulation effects were compared for the sequences with burst lengths of 10 s (TXL_10_50), 20 s (TXL_20_40), and 40 s (TXL_40_40). The burst was repeated six times at intervals of 50, 40, and 40 s for TXL_10_50, TXL_20_40, and TXL_40_40, respectively (Figure 1). For TXL_10_50, the frequency of the discharge peaks increased in the EEG waveform (**Figure** **3**A). The number of epileptic spikes with TXL_10_50 was statistically comparable with that in the PTZ‐control group at the post‐stimulation periods from 10AS to 30AS (*p* = 0.435, 0.354, and 0.435 at 10AS, 20AS, and 30AS, respectively; two‐tailed Mann–Whitney U test). As the stimulation length elongated from 10 to 40 s (TXL_10_50, TXL_20_40, TXL_40_40), the number of epileptic spikes decreased from 2.63, 1.94, 1.07 times the baseline, respectively, at 10AS while the difference in the number of epileptic spikes ratio between that of the PTZ‐control was only significant in the TXL_40_40 group (*p* = 0.435, 0.943 and 0.030 at 10AS for TXL_10_50, TXL_20_40, and TXL_40_40, respectively; two‐tailed Mann–Whitney U test) (Figure 3B,C). In the delta band, the spectral power of the EEG for the TXL_10_50 condition was comparable to that for the PTZ‐control group (*p* = 0.524 at 30AS; two‐tailed Mann–Whitney U test) while that of the EEG for TXL_40_40 condition was lower than that for the PTZ‐control group. (*p* = 0.045 at 30AS; two‐tailed Mann–Whitney U test) (Figure 3D). In the theta band, the spectral power of the EEG for the TXL_10_50 condition was comparable to that for the PTZ‐control group (*p* = 0.524 at 30AS; two‐tailed Mann–Whitney U test). However, the trend of reduction in the theta power was significant as the burst length was increased to 20 and 40 s (*p* = 0.171 and 0.019 at 30AS for TXL_20_40 and TXL_40_40, respectively, compared to PTZ‐control; two‐tailed Mann–Whitney U test) (Figure 3D). In the CBV curve, with TXL_10_50, Δ[Hb] was sharply increased by + 12.89% at 20AS compared to that in the baseline and was maintained at a higher level than that in the PTZ‐control group (*p* = 0.699 at 20AS; two‐tailed Mann‐Whitney U‐test), while the CBV with TXL_40_40 was similar to that of the normal control (*p* = 0.699 at 20AS; two‐tailed Mann–Whitney U‐test) (Figure 3E).

### 2.3 Upregulation and Downregulation of Epileptiform Activities Selected by Adjusting the Interval Between Bursts in the rtFUS Sequence

**Figure 4:**
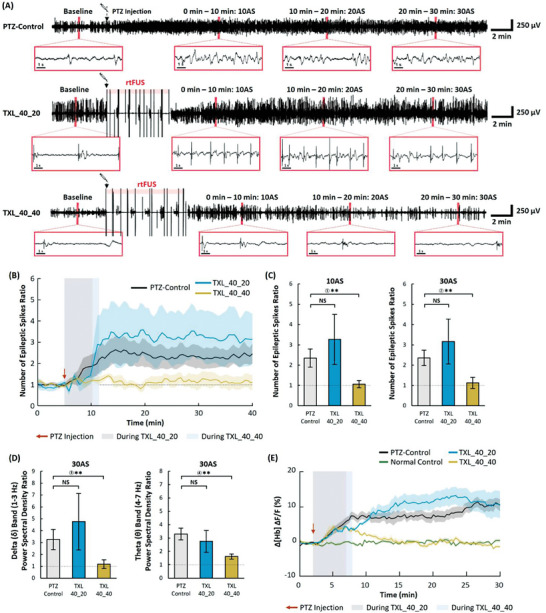
Changes in epileptiform activities during and after TXL_40_20 and TXL_40_40. A) Time courses of EEG signals in the PTZ‐control and stimulated experimental groups. B) Time courses of the number of epileptic spikes ratio in each group (PTZ‐control: *n* = 8, TX groups: *n* = 5). C) Ratio of the number of epileptic spikes measured at time periods of 0–10 min (10AS) and 10–20 min (20AS) (PTZ‐control: *n* = 8, TX groups: *n* = 5). D) Power spectral density ratio of EEG signals at the delta (0.5–3 Hz) and theta (4–7 Hz) frequency bands (PTZ‐control: *n* = 8, TX groups: *n* = 5). E) Changes in the cerebral blood volume [Hb] calculated from 530 nm reflectance data (PTZ‐control: *n* = 6, TX groups: *n* = 6). Data presented as mean ± SEM, *p*‐values calculated via two‐tailed Mann–Whitney U test, ** : *p* <0.05, * : *p* <0.10, NS : *p*> 0.10. *p* = ① 0.030 ② 0.030 ③ 0.045 ④ 0.019.

The epileptiform activity changes by TXL_40_40 were compared with that by TXL_40_20, which also had a burst length of 40 s; however, the interval between the bursts was reduced from 40 to 20 s (Figure 1). TXL_40_20 stimulation demonstrated rebound excitatory effects in the post‐stimulus period (**Figure** **4**A). TXL_40_20 generated a similar number of epileptic spikes as that in the PTZ‐control group (*p* = 0.943, 0.833, and 0.943 at 10AS, 20AS, and 30AS, respectively; two‐tailed Mann–Whitney U test). In contrast, TXL_40_40 stimulation demonstrated prominent suppressive effects both during and after stimulation (Figure 4B,C). The spectral density in the delta band was higher with TXL_40_20 at 30AS than that in the PTZ control. However, the difference was not significant owing to large deviations with TXL_40_20 (*p* = 0.833; two‐tailed Mann–Whitney U test) (Figure 4D). While the CBV response of TXL_40_20 was similar to that of TXL_40_40 during the stimulation period, the Δ[Hb] sharply increased after TXL_40_20, unlike the CBV responses after TXL_40_40, ultimately becoming higher than that of the PTZ control (Figure 4E).

### 2.4 Transformation from Suppressive to Excitatory States Through a Hybrid Sequence

**Figure 5:**
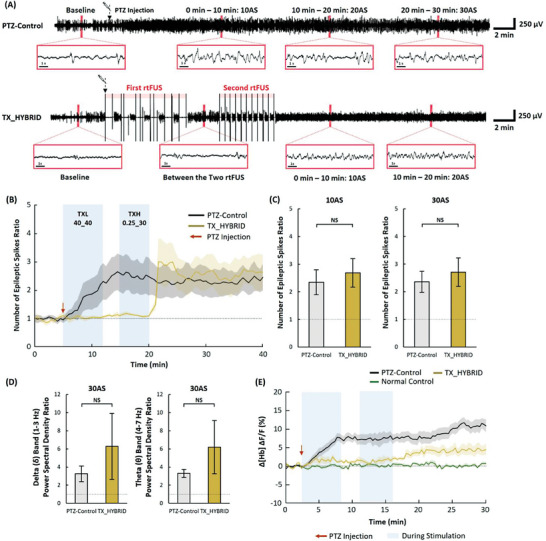
Changes in epileptiform activities during and after TX_HYBRID (consecutive transmits of TXL_40_40 and TXH_0.25_30). A) Time courses of EEG signals in the PTZ‐control and the stimulated experimental groups. B) Time courses of the number of epileptic spikes ratio in each group (PTZ‐control: *n* = 8, TX groups: *n* = 5). C) Ratio of the number of epileptic spikes measured at time periods of 0–10 min (10AS) and 10–20 min (20AS) (PTZ‐control: *n* = 8, TX groups: *n* = 5). D) Power spectral density ratio of EEG signals at the delta (0.5–3 Hz) and theta (4–7 Hz) frequency bands (PTZ‐control: *n* = 8, TX groups: *n* = 5). E) Changes in the cerebral blood volume [Hb] calculated from 530 nm reflectance data (PTZ‐control: *n* = 6, TX groups: *n* = 6). Data presented as mean ± SEM, *p*‐values calculated via two‐tailed Mann–Whitney U test, ** : *p* <0.05, * : *p* <0.10, NS : *p*> 0.10.

As shown in previous studies, TXL_40_40 demonstrated strong suppressive effects. In contrast, the insignificant suppressive effects observed with TXH_0.25_30 – that showed suppressive effects with low statistical significance [19.91% less epileptic spikes ratio (*p* = 0.724), 16.61% less delta PSD (*p* = 0.833) and 38.79% less theta PSD (*p* = 0.093) than that of the PTZ‐control at 30AS]. Although the single use of TXL_40_40 maintained the suppressive effect for more than 30 min, the epileptiform activities were sharply transformed into the excitatory state with the use of TXH_0.25_30, followed by a 3 min resting time after TXL_40_40 (TX_HYBRID, **Figure** **5**A). The number of epileptic spikes ratio in the TX_HYBRID group was always comparable with the PTZ‐control group across all post‐stimulation periods at 10AS, 20AS, and 30AS (*p* = 0.943, 0.943, and 0.622, respectively; two‐tailed Mann–Whitney U test) (Figure 5B,C). The elevations of the power spectral densities in the delta and theta band after TX_HYBRID (30AS) were comparable to that of the PTZ‐control group (Figure 5D) (*p* = 1.000 and 0.622 at 30AS, respectively; two‐tailed Mann–Whitney U test). Regarding CBV changes, the Δ[Hb] was reduced after TXL_40_40 sonication, whereas TXH_0.25_30 increased the blood volume, approaching that of the PTZ control (Figure 5E).

### 2.5 Immunohistochemistry Results

**Figure 6:**
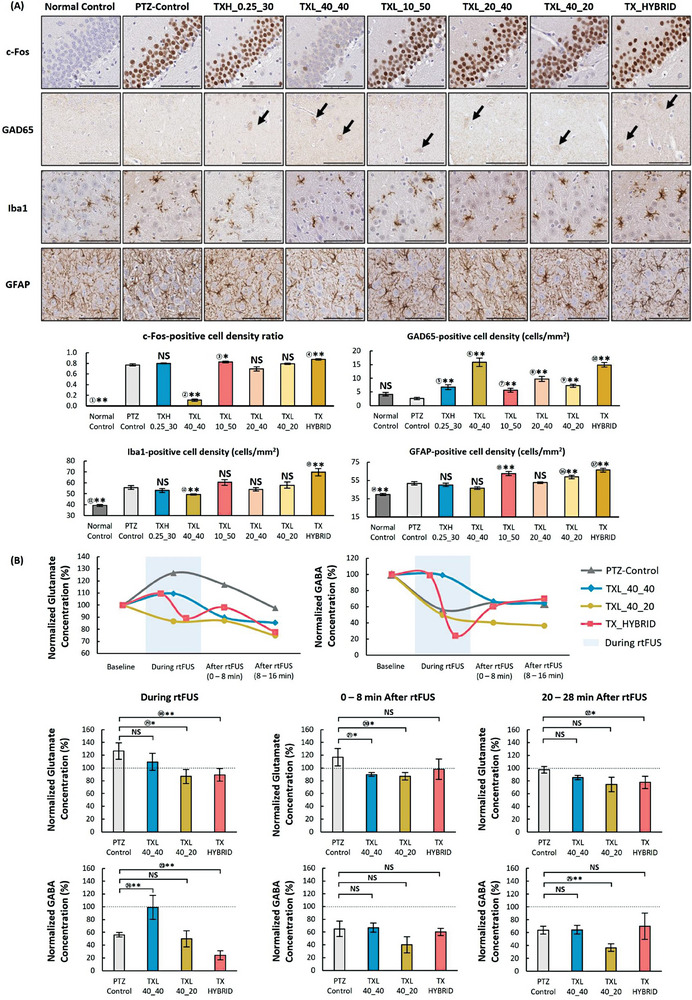
Immunohistochemistry results and multitemporal neurotransmitter analysis from microdialysis. A) Representative slice images and quantitative measurements from c‐Fos‐, GAD65‐, Iba1‐, and GFAP‐stained brain tissue at the dentate gyrus. Quantitative measurements were made in the stained cell ratio for c‐Fos and the stain‐positive cell density for GAD65, Iba1, and GFAP (PTZ‐control: *n* = 6, TX groups: *n* = 6). B) Changes in the normalized glutamate and GABA concentration (%) during and after TXL_40_40, TX_40_20 and TX_HYBRID compared to the baseline (PTZ‐control: *n* = 8, TX groups: *n* = 5). Data presented as mean ± SEM, *p*‐values calculated via two‐tailed Mann–Whitney U test, ** : *p* <0.05, * : *p* < 0.10, NS : *p*> 0.10. *p* = ① 0.002 ② 0.002 ③ 0.093 ④ 0.004 ⑤ 0.004 ⑥ 0.002 ⑦ 0.015 ⑧ 0.002 ⑨ 0.002 ⑩ 0.002 ⑪ 0.002 ⑫ 0.009 ⑬ 0.009 ⑭ 0.002 ⑮ 0.004 ⑯ 0.041 ⑰ 0.002 ⑱ 0.048 ⑲ 0.073 ⑳ 0.062 ㉑ 0.088 ㉒ 0.073 ㉓ 0.003 ㉔ 0.030 ㉕ 0.018.

**Figure** **6**A shows the immunohistochemical results of the samples harvested from sacrificed animals immediately after each experiment. While c‐Fos‐expressed cells were rarely found in the normal control group, c‐Fos‐labeled cells were abundant in the PTZ‐control group (*p* = 0.002; two‐tailed Mann–Whitney U test). The increase of the proportion of c‐Fos‐positive cells in the TXL_10_50 group was trending toward significance to that in the PTZ‐control group (*p* = 0.093; two‐tailed Mann–Whitney U test). As shown in the bar graph, 82.58% (standard error of the mean [SEM] = 1.83%) of neuronal cells expressed c‐Fos with TXL_10_50, and 77.14% (SEM = 2.12%) of cells expressed c‐Fos in the PTZ‐control group. TXL_20_40 (*p* = 0.240; two‐tailed Mann–Whitney U test), TXL_40_20 (*p* = 0.394; two‐tailed Mann–Whitney U test) and TXH_0.25_30 (*p* = 0.394; two‐tailed Mann–Whitney U test) demonstrated c‐Fos‐positive cell ratios comparable to those of the PTZ‐control group. Furthermore, TX_HYBRID presented an even higher value of ≈87.47% (SEM = 1.25%) than that of PTZ‐control (*p* = 0.004; two‐tailed Mann–Whitney U test). In contrast, the expression ratio was drastically reduced to 11.11% with TXL_40_40 compared with the PTZ‐control group (*p* = 0.002; two‐tailed Mann‐Whitney U test).

Although the normal and PTZ‐control cases barely exhibited glutamic acid decarboxylase 65‐kilodalton isoform (GAD65)‐positive cells, the sonicated conditions exhibited a greater number of GAD65‐expressed cells. The GAD65‐positive cell density with TXL_40_40 (mean = 15.88 cells mm^−2^; SEM = 1.53 cells mm^−2^) was ≈6.2‐fold higher than that in the PTZ‐control group (mean = 2.56 cells mm^−2^; SEM = 0.42 cells mm^−2^). Although sharing the same burst duration, TXL_40_20 exhibited a lower expression of GAD65‐positive cells (mean = 7.27 cells mm^−2^; SEM = 0.61 cells mm^−2^) than that of TXL_40_40 (mean = 15.88 cells mm^−2^; SEM = 1.53 cells mm^−2^), while both groups showed statistically significant difference compared to the PTZ‐control (*p* = 0.002 and 0.002, respectively; two‐tailed Mann–Whitney U test). The GAD65‐expressed cell densities for TXL_10_50 (mean = 5.60 cells mm^−2^; SEM = 0.71 cells mm^−2^) and TXL_40_20 (mean   7.27 cells mm^−2^; SEM = 0.61 cells mm^−2^) were lower than the cell density with TXL_40_40 (mean = 15.88 cells mm^−2^; SEM = 1.53 cells mm^−2^) and TX_HYBRID (mean = 14.89 cells mm^−2^; SEM = 0.81 cells mm^−2^).

While the ionized calcium‐binding adaptor molecule 1 (Iba1)‐expressed cell density in the normal control group was 39.30 cells mm^−2^ (SEM = 0.89 cells mm^−2^), the number increased to 55.62 cells mm^−2^ (SEM = 1.79 cells mm^−2^) in the PTZ‐control group. The density of activated cells with TXL_40_40 was 49.36 cells mm^−2^ (SEM = 0.59 cells mm^−2^), which was significantly lower than that of the PTZ‐control group (*p* = 0.009, two‐tailed Mann–Whitney U test). The Iba1‐expressed cell densities in TXH_0.25_30, TXL_10_50, TXL_20_40 and TXL_40_20 were 54.13 cells mm^−2^ (SEM = 1.68 cells mm^−2^), 60.50 cells mm^−2^ (SEM = 2.61 cells mm^−2^), 54.07 cells mm^−2^ (SEM = 1.58 cells mm^−2^) and 57.91 cells mm^−2^ (SEM = 2.95 cells mm^−2^), respectively. However, those values were not statistically different (*p* = 0.394, 0.240, 0.699, 0.589 for TXH_0.25_30, TXL_10_50, TXL_20_40 and TXL_40_20, respectively, two‐tailed Mann–Whitney U test) from the one with PTZ‐control. TX_HYBRID induced the highest Iba1‐expression level at 69.72 cells mm^−2^ (SEM = 3.51 cells mm^−2^) among all the experimental groups, and the increase was significant compared with PTZ‐control group (*p* = 0.009, two‐tailed Mann–Whitney U test).

In the glial fibrillary acidic protein (GFAP) responses indicating astrocytic reactivity, GFAP density increased to 51.76 cells mm^−2^ (SEM = 1.96 cells mm^−2^) in the PTZ‐control group, unlike the normal control group, which presented a density of 39.71 cells mm^−2^ (SEM = 1.23 cells mm^−2^) (*p* = 0.002; two‐tailed Mann–Whitney U test). Furthermore, the GFAP‐positive cell densities of TXL_10_50, TXL_20_40, and TXL_40_40 gradually decreased to 62.58 (SEM = 2.52), 52.59 (SEM = 0.82), and 47.30 cells mm^−2^ (SEM = 1.82 cells mm^−2^), respectively, while the GFAP‐positive cell densities with TXL_20_40 and TXL_40_40 were comparable with that with PTZ‐control (*p* = 0.004, 0.937 and 0.132, respectively, compared to that of PTZ‐control; two‐tailed Mann–Whitney U test). In contrast, TXL_40_20 demonstrated a higher density of 58.85 cells mm^−2^ (SEM = 2.06 cells mm^−2^) than that of PTZ‐control (*p* = 0.041; two‐tailed Mann–Whitney U test). The degree of GFAP expression with TXH_0.25_30 was similar to that with PTZ‐control (*p* = 0.485; two‐tailed Mann–Whitney U test), demonstrating a reduced level of 50.18 cells mm^−2^ (SEM = 2.08 cells mm^−2^). TX_HYBRID presented a higher GFAP‐density level of 66.43 cells mm^−2^ (SEM = 2.36 cells mm^−2^) than the PTZ‐control group (*p* = 0.002; two‐tailed Mann–Whitney U test).

### 2.6 Multitemporal Neurotransmitter Analysis Through Microdialysis

Changes in neurotransmitters through bidirectional neuromodulation were measured using proteomic analysis of ISF samples at baseline and 0, 8, and 16 min after sonication (Figure S2A–C, Supporting Information). In hybrid transmission, an additional ISF sample was acquired between the two transmissions. For comparison, the changes in the neurotransmitter levels were divided by the baseline values. In the PTZ‐control group, the levels of glutamate were observed to be increased, while that of GABA was observed to be decreased (Figure 6B) compared to the baseline levels after PTZ injection. In contrast, the use of TXL_40_40 significantly reversed the pattern of GABA concentration changes observed in the PTZ‐control (*p* = 0.030; two‐tailed Mann–Whitny U test) while a decrease in glutamate was not significant compared with that in the PTZ‐control group (*p* = 0.530; two‐tailed Mann–Whitney U test). After the stimulation, GABA levels became similar with that in the PTZ‐control group (*p* = 0.343 and *p* = 1.000 at 0 – 8 min and 20 – 28 min after rtFUS, respectively; two‐tailed Mann–Whitny U test). The glutamate concentration decreased during TXL_40_20 was trending toward significance (*p* = 0.073 compared to that of PTZ‐control; two‐tailed Mann–Whitny U test) while the GABA decrease during TXL_40_20 application was not significant (*p* = 0.202 compared to that of PTZ‐control; two‐tailed Mann–Whitny U test). However, the GABA concentration in TXL_40_20 group at 20–29 min after rtFUS dropped significantly compared to that in the PTZ‐control group (*p* = 0.018, compared to that of the PTZ‐control; two‐tailed Mann–Whitny U test) while glutamate levels were similar between two groups. With hybrid transmission, both GABA and glutamate levels became lower during the second transmission (TXH_0.25_30) than PTZ‐control (*p* = 0.030 and 0.048 for GABA and glutamate, respectively, compared to that of the PTZ‐control; two‐tailed Mann–Whitny U test), but immediately became similar with that in PTZ‐control group (*p* = 0.876 and 0.343 for GABA and glutamate, respectively, compared to that of the PTZ‐control; two‐tailed Mann–Whitny U test).

## 3 Discussion

In previous studies, tFUS that sonicated a brain region a single time was more or less effective in lowering epileptiform activities. In the current study, rtFUS, which involves the repetitive transmission of elongated bursts, was first introduced. In contrast to tFUS, rtFUS was able to fully suppress epileptic discharge, resulting in a state comparable to baseline brain responses as measured by EEG and CBV. Surprisingly, the c‐Fos response in the IHC study was barely expressed in the brain samples receiving TXL_40_40 stimulation, whereas the response rate of GAD65 was highest with TXL_40_40 that repeated to transmit a 40 s long low‐intensity burst six times with an interval of 40 s. In microdialysis studies, GABA levels during stimulation with TXL_40_40 were prominently increased, and this observation could support the highest number of GAD65 stains after stimulation with TXL_40_40. Meanwhile, the use of a single transmission of a 40 s long burst suppressed epileptiform activities for a brief period and eventually resulting in symptoms similar to those of the PTZ‐control group. These results suggested that repetitive ultrasonic stimulation with elongated bursts can provide reliable and sustainable suppressive effects by modulating GABA release.

In contrast to TXL_40_40, which induced seizure‐suppressive effects, shorter bursting intervals of 20 s (TXL_40_20) could exacerbate epileptiform activities, as shown by EEG, CBV, and IHC. The interval between ultrasound stimulation might not be sufficient for 20 s and needs to be longer than 40 s to maintain a reasonable GABA level for suppression. This is supported by previous findings that reported an excretion time for GABA with electrical stimulation exceeding 40 s in a mouse model.^[^ ^36^ ^]^ This assumption was verified by performing multitemporal measurements of neurotransmitters because the GABA level after TXL_40_20 was continuously decreased and eventually became significantly lower than that with PTZ‐control (Figure 6B). Furthermore, in immunohistochemical studies, the number of c‐Fos‐expressing cells was reduced by 85.6% in samples treated with TXL_40_40 compared to the PTZ‐control. In comparison, GAD65 expression increased by 520% compared to that in the PTZ‐control group (Figure 6A). However, with TXL_40_20, the expression of GAD65 was significantly reduced by 54.2% compared to that with TXL_40_40 whereas c‐Fos expression was comparable to that in the PTZ‐control group.

Our results further demonstrated that burst pressure and length also influenced these suppressive effects. rtFUS using a sequence repeating a shorter (0.25 s) but stronger pressure (1 MPa) burst at every 30 s for eleven times (TXH_0.25_30) demonstrated only mild antiepileptic effects. Considering previous studies that reported neuronal activation with high‐pressure stimulation, TXH_0.25_30 may simultaneously activate excitatory and inhibitory neurons and reduce the net suppressive effect. As shown in the IHC results, the expression ratio of GAD65 with TXH_0.25_30 was 2.74 times higher than that of the PTZ‐Control, while the expression levels of c‐Fos, Iba‐1 and GFAP with TXH_0.25_30 were comparable with those in the PTZ‐control. Interestingly, TXH_0.25_30 induced an excitatory response when the transmit sequence was applied after TXL_40_40. Therefore, controlling the progressive direction of epileptiform activities with short‐ and high‐intensity stimulation was challenging. In contrast, burst lengths longer than 40 s clearly demonstrated seizure suppressive effects. c‐Fos expression decreased drastically, and GAD65 expression gradually increased as the burst length increased from 10 to 40 s with low stimulation pressure levels. These results indicated that the burst pressure and length of rtFUS could be key parameters in determining the progressive direction of epileptiform activities in animal models of drug‐induced epilepsy.

In addition to generating different epileptiform activities for different burst lengths or intervals in rtFUS, the suppressive effects could be cleared and translated to excitatory states, worsening epileptic activity by hybrid transmission. The application of TX_HYBRID, comprising a back‐to‐back transmission of TXL_40_40 and TXH_0.25_30 with a 3 min interval, induced the transformation of the suppressant effect into a paradoxically proconvulsant cortical response (Figure 5B), although TXL_40_40 and TXH_0.25_30 alone induced strong and mild suppressive effects (Figure 2B). In the IHC experiments, the expression rates of GAD65 and c‐Fos with TX_HYBRID were higher than those in PTZ‐control. In the multitemporal neurotransmitter analysis, the elevated GABA level induced by TXL_40_40 also immediately decreased after TXH_0.25_30 treatment, becoming similar to that of the PTZ‐control. Therefore, both inhibitory and excitatory responses were induced by highly conjugated and compensatory regulation, which may also be explained by an increase in GAD65 and c‐Fos expression. Amplified excitation by TX_HYBRID also induced more extensive hippocampal damages than the responses in other animals receiving sole stimulation types. The numbers of both Iba1‐ and GFAP‐expressing cells were the highest in the TX_HYBRID group compared to that in the PTZ‐control group.

Although the EEG and CBV measurement of brain activities for temporal lobe epilepsy were not located around the hippocampus, but mostly in the prefrontal cortex, the measured signals should reflect deep brain activities because of the prefrontal limbic network connection to deep brain structures.^[^ ^37^ ^]^ Because a four‐channel EEG can measure both the frontal and midbrain regions in both hemispheres, a simple topography revealing the number of discharge peaks could be acquired by linear interpolation among the numbers from the four channels. As shown in the Supporting Information figure (Figure S3, Supporting Information) and videos, the excitatory brain response to PTZ injection was initially identified in the prefrontal region, and the signal was stronger than that in the midbrain region. Furthermore, the GABA and glutamate levels measured in the deep brain region using microdialysis demonstrated temporal patterns similar to epidural electrical signal changes. In future work, deep brain electrode recordings may help acquire direct proof of the seizure modulation capability of rtFUS.

The number of Iba1‐positive cells, which are reactive near‐damaged neuronal cells sharply increased in the PTZ control group compared to that of the normal control group. The amount of activation increased with TX_HYBRID and decreased with TXL_40_40 compared with PTZ‐control. Therefore, rtFUS sequences that induce more frequent EEG discharges could induce higher cellular damage than that of other sequences. In contrast, other sequences suppressing epileptiform activities could preserve neuronal cells, although they could not fully recover from the damage caused by suppressive treatments. To confirm that the damage to neuronal cells was caused by the proconvulsant effect and not by the delivery of ultrasonic energy, Cresyl Violet staining was performed on the normal control and PTZ‐/rtFUS+ groups. The Nissl‐stained brain slice images demonstrated no damage to the rat brain after TXH_0.25_30 and TXL_40_40 stimulations, which may be the worst cases for sonication amplitude and time, respectively (Figure S4, Supporting Information).

Bidirectional neuromodulation was achieved by adjusting the acoustic parameters of stimulation strength, length, and the interval of stimulus repetition. For instance, post‐stimulus extracellular GABA level was lowered by shorter intervals of longer stimulation length (TXL_40_20) that might cause GABA depletion because post‐stimulus GABA release took tens of seconds to occur, as demonstrated in a prior study using electrical stimulation^[^ ^24^ ^]^ and the stimulation before the GABA recovery might reduce extracellular GABA. Meanwhile, a longer interval (TXL_40_40) could elevate extracellular GABA by allowing sufficient post‐stimulus GABA recovery time. Although we demonstrated the changes in GABA over time using protein analysis with microdialysis, further analysis using a device that presents the changes in GABA with higher time resolution, such as an implantable electrochemical GABA sensor,^[^ ^36^ ^]^ may be needed to prove the depletion and recovery of post‐stimulus GABA over time. Moreover, molecular mechanistic studies are required to extend bidirectional control to other applications. A study has shown that the astrocytic mechanosensitive TRPA1 channel plays a significant role in inducing neuronal activation and eventually releasing glutamate by low‐intensity ultrasound stimulation,^[^ ^38^ ^]^ while there are only limited molecular mechanistic studies investigating pathways of GABA release in response to ultrasound stimulation. The navigation of possible pathways that activate GABA excretion with ultrasound stimulation should be planned to clearly understand bidirectional neuromodulation.

We demonstrated the effectiveness of rtFUS in modulating acutely elevated neuronal responses in a chemically induced epilepsy model. As both anticonvulsive and proconvulsive effects were immediately acquired and sustained for at least 30 min using low‐intensity rtFUS, the pulse sequence may be applied to treat epilepsy in the clinic. To achieve this, a seizure detection system, such as a closed‐loop system with EEG, will need to be combined with rtFUS to rapidly suppress signals by sonication as soon as epileptiform activities are detected. Furthermore, this technology may be applicable to other neuronal disorders originating from a dysfunction in the control of neurotransmitters, such as depression and drug addiction.^[^ ^39^, ^40^, ^41^, ^42^ ^]^ As introduced in a previous review,^[^ ^43^ ^]^ ultrasound neuromodulations with tFUS are highlighted to be effective in brain therapeutics while the efficacies in some results were mild. The use of different ultrasound pulse sequences modulates different outcomes in treating Alzheimer's disease.^[^ ^44^ ^]^ Therefore, we expect that the efficacies of ultrasound neuromodulation can be amplified and prolonged by using rtFUS.

## 4 Conclusion

In our study, the bidirectional modulation of epileptiform brain activity was first demonstrated by changing the burst duration, interval, and amplitude of rtFUS. A stimulation train repeating elongated bursts with sufficiently long intervals was shown to effectively suppress epileptiform activities. In contrast, stimulation transmitting the same burst length with a shorter interval worsened the epileptic responses. Furthermore, the suppressive effect achieved by the long burst could be reversed using a short, strong burst train, leading to a shift from a suppressive to an excitatory state. The IHC and proteomic analyses conducted in this study allowed us to conclude that bidirectional modulation of epileptiform activity with rtFUS was achieved by controlling the excretion of GABA in sonicated brain tissue.

## 5 Experimental Section

All animal experiments were performed in accordance with the Korean Ministry of Food and Drug Safety Guide for the Care and Use of Laboratory Animals and were conducted according to protocols approved by the Institutional Animal Care and Use Committees (IACUC). This study was reviewed and approved by the IACUC of Samsung Biomedical Research Institute (SBRI) and WOOJUNG BIO (Approval Numbers: SBRIIACUC20200109003 and IACUC2301‐025). SBRI and WOOJUNG BIO are Association for Assessment and Accreditation of Laboratory Animal Care International (AAALAC International) accredited facility and abide by the Institute of Laboratory Animal Resources (ILAR) guide.

### Animal Preparation and Epileptic Induction

Fifty‐nine 8‐week‐old male Sprague–Dawley rats weighing 300–320 g (Orient Bio, Inc.) were used in the experiments. For the EEG, eight rats were chosen for the non‐stimulated PTZ+/tFUS‐control (PTZ‐control) group, and five rats were chosen for each stimulated experimental group. IHC was performed within the EEG group for five rats in each of the PTZ‐control and stimulated experimental groups, and five additional rats were evaluated as the PTZ‐/tFUS‐control (normal control) group. For wide‐field optical imaging, six rats each from the PTZ control, normal control, and stimulated experimental groups were evaluated. For the microdialysis, eight rats were chosen for PTZ‐control, and five rats were chosen for each group of TXL_40_40, TXL_40_20 and TX_Hybrid. (**Table** **1**) Animals were anesthetized using urethane (1.25 g kg^−1^ body weight in 1.25 g 4 mL^−1^ saline; Sigma–Aldrich, U‐2500‐500G) and pentylenetetrazol (100 mg kg^−1^ body weight in 10 mg 0.1 mL^−1^ saline; Sigma–Aldrich, P6500‐25G), administered acutely and intraperitoneally to induce epileptic seizures.

**Table 1:** Animal Allocation for Each Experimental Group and Studies.

	Normal Control	PTZ‐Control	TXH_0.25_30	TXL_10_50	TXL_20_40	TXL_40_40	TXL_40_20	TX_HYBRID
EEG/IHC^a)^	n = 0/6	n = 8/6	n = 5/6	n = 5/6	n = 5/6	n = 5/6	n = 5/6	n = 5/6
CBV	n = 6	n = 6	n = 6	n = 6	‐	n = 6	n = 6	n = 6
Microdialysis	‐	n = 8	‐	‐	‐	n = 5	n = 5	n = 5

^a)^ Immunohistochemical analyses were performed with n = 6 for each staining method. Additionally, the safety of focused ultrasound stimulation was evaluated in the PTZ‐/rtFUS+ group (n = 3), where TXH_0.25_30 was applied to the left hemisphere and TXL_40_40 was applied to the right hemisphere (Figure S4, Supporting Information).

### Ultrasound Transducer Fabrication

A lead zirconate titanate (Del Piezo Specialties LLC., DL‐47) 690 kHz single‐element ring transducer with inner and outer diameters of 10 and 31 mm, respectively, was fabricated with a focal distance of 45 mm and a bandwidth of 25%. An acoustic coupling gel‐filled cone‐shaped beam guide was attached to the front side of the transducer. The beam was focused 5 mm ahead of the tip of the collimator with −3 dB beam sizes of 4.7 and 2.2 mm along the axial and lateral directions, respectively (Figure S1A, Supporting Information). By placing the tip of the transducer above the parietal bone, the acoustic beam was delivered to the right anterior thalamic nuclei (ATN) (AP = −1.5 mm, ML = + 1.5 mm, DV = 5.0–5.5 mm; AP denotes the anterior (+) or posterior (−) distance from the bregma; ML denotes the lateral distance from the bregma; and DV denotes the dorsoventral distance from the bregma). The beam pathway was confirmed by overlaying the beam profile on the Paxinos rat brain atlas^[^ ^45^ ^]^ along the sagittal and coronal planes (Figure S1B,C, Supporting Information).

### Sonication and Monitoring Protocol

The stimuli were classified into two types to simplify the ultrasonic conditions and mimic electrical stimulations generating rebound excitations: one with a low‐pressure (0.25 MPa) consecutive burst train (TXL sequences), and the other with shorter, high‐pressure (1.0 MPa) bursts (TXH sequence) with a 50% duty cycle of 100 Hz pulse repetition frequency. TX_HYBRID, a back‐to‐back transmission of TXL_40_40 and TXH_0.25_30 was generated assuming that rebound excitation could be artificially induced using brief excitation followed by inhibitory stimulation.

Figure S1D (Supporting Information) depicts the entire experimental platform for delivering ultrasonic energy to the target region and recording EEG signals. The rats were anchored to a custom‐made stereotaxic frame (Scitech Korea, Inc.), and ultrasound pulsation was generated using two function generators. The first function generator (Tektronix, AFG3252) sent out a pulse train of 100 Hz, and the second function generator (Agilent, 33250A) transmitted a 5 ms, 3450 cycle, 690 kHz burst to secure a 50% duty cycle (Figure S1D, Supporting Information) at every rising edge in the pulse train from the first function generator. The generated waveform was then passed through a radiofrequency power amplifier (Electronics and Innovation, 350 L) and applied to the transducer. The experimental protocol involved: 1) anesthesia; 2) pre‐tFUS (baseline) monitoring; 3) PTZ injection and sonication; 4) post‐tFUS monitoring; and 5) euthanization and brain tissue harvesting. Figure 1 shows the overall timeline of sonication and data acquisition.

### EEG Recording

The rats were stereotactically implanted with five cortical screw electrodes (Fine Science Tools, 19010‐00 screws) to record the EEG signals (Figure S1A, Supporting Information). The implantation coordinates were adopted based on a stereotactic atlas^[^ ^45^, ^46^ ^]^: C4/C3 (primary motor cortex; A = + 2.2 mm, L = ± 3.2 mm) and T6/T5 (temporal association cortex; A = −8.3 mm, L = ± 5.8 mm). A reference ground electrode was implanted on the posterior side of the lambda. EEG was performed to monitor changes in neuronal activity using a four‐channel commercial EEG acquisition system (Natus, NicoletOne vEEG) at a sampling rate of 500 Hz. The ratio of the number of epileptic spikes at baseline to the number at specific time points was calculated to assess the epileptic severity. Epileptic spikes were defined as local maximum points with amplitudes greater than twice the standard deviation from individual baseline EEG activities lasting 70 ms. The discharge peaks of the EEG signal were counted every 30 s in a 1 min segment across the monitoring period and averaged every 10 min for each animal. The power spectral density values were computed for every 1 min segment using Welch's method and also averaged every 10 min for each animal. Pairwise comparison was made at each time period for the statistical analysis where each value represented a single value from a single animal.

### Immunohistochemistry

IHC was performed using c‐Fos, GAD65, Iba‐1, and GFAP staining of rat brain sections. The rats were euthanized after the EEG recording and transcardially perfused with saline to obtain brain tissue samples. The ratio of c‐Fos‐positive cells to total cells in the granular layer of the dentate gyrus was quantified using ImageJ software (National Institutes of Health, 1.53c) to compare the degree of excitatory responses generated under ultrasonic stimulus conditions. To compare inhibitory synaptic transmission, GAD65‐positive cell densities were measured in the molecular and granular layers of the dentate gyrus. Staining for Iba1 and GFAP was performed to investigate the reactivity of microglia and astrocytes, respectively, by computing the Iba1‐ and GFAP‐positive cell densities. All values were measured three times independently from a different person in a random order to avoid biased erroneous measurement and averaged in a single value for each animal. A pairwise comparison was made where each value represented a single value from a single antibody‐stained brain slice from a single animal.

### Cerebral Blood Volume Measurement

Craniotomies were performed on isoflurane‐anesthetized rats secured in a custom‐made stereotaxic frame. The cranial window region was created above the left ATN (A = −1.5 mm, L = −1.5 mm), such that the rtFUS stimulus could be applied on the right ATN while simultaneously imaging the cranial window (Figure S2C,D, Supporting Information). After an overnight recovery, urethane (1.25 mg kg^−1^ body weight) was injected intraperitoneally for wide‐field optical imaging.

Optical measurement was conducted for the normal control, PTZ‐control, and tFUS‐stimulated groups to examine the regional hemodynamic response of cerebral blood volume (CBV). The imaging system consisted of a macro‐zoom microscope (Olympus, MVX10), an sCMOS camera (Andor, Zyla 5.5), and a light‐emitting diode source (Figure S2C, Supporting Information). For the experiment, raw reflectance data were collected under green (530 nm) light, as 530 nm is primarily sensitive to changes in the local total blood volume, denoted as the HbT.^[^ ^47^, ^48^ ^]^ Each imaging trial was recorded for 30 min, including a 2 min baseline (pre‐PTZ/tFUS).

### Microdialysis

Microdialysis analysis was conducted to determine the amount of neurotransmitter secreted during the alterations in epileptiform activities generated by the rtFUS transmission sequences (Figure S2A, Supporting Information) to demonstrate the cause of bidirectional modulation in animal models of drug‐induced epilepsy. The guide cannula was implanted in twenty male Sprague‐Dawley rats weighing 300–320 g (Koatech, Inc.) under respiratory anesthesia using a hand drill at the target coordinates of the hippocampal region CA2/CA3 (Coordinates −3.80 mm posterior (AP), −4.40 mm lateral (ML), and −2.20 mm ventral (DV), 26 degrees lateral) (Figure S2B, Supporting Information). After the guide cannula reached the target coordinates, dental cement was applied for fixation, avoiding the position of the ultrasound transducer, and the incision site was sutured with a silk suture for the recovery period. For proteomic analysis, five rats were chosen for the PTZ‐control group (PTZ+/tFUS‐) and five rats were chosen for each experimental group: TXL_40_40, TXL_40_20, and TX_HYBRID.

Two days after the guide cannula insertion surgery, the recovered rats were intraperitoneally anesthetized with urethane (1.25 g kg^−1^ body weight in 1.25 g 4 mL^−1^ saline; Sigma–Aldrich, U‐2500‐500G). After the anesthetized rats were secured to the stereotaxic frame, the dummy was removed from the guide cannula and a CMA12 1‐mm membrane probe (CMA Microdialysis) was inserted. A 5 mL glass syringe filled with aCSF (M dialysis AB) was mounted on a syringe pump (KD Scientific) and connected to the probe inlet. The tubing was connected to the probe outlet to collect the interstitial fluid (ISF) in a sample collector (BASi, U.S.A). After ≈1 h of stabilization (1 point 8 min^−1^), Interstitial fluid (ISF) was collected by sampling for 1 h. For microdialysis, samples were collected for 8 min at a flow rate of 1.3 µL min^−1^ in order to obtain a sufficient amount (minimum 10 µL) of ISF for the neurotransmitter analysis. The sample collection time was minimized to match the sonication duration (440 s/340 s), but due to the fixed flow rate and the minimum required collection volume, the sample collection time was inevitably longer than the sonication duration. Therefore, a portion of the baseline (40 s for TXL_40_40 and 140 s for TXL_40_20) was collected together when analyzing the neurotransmitter concentration during the rtFUS. All samples were stored in a freezer at −80 °C.

Neurotransmitter analysis of the microdialysis samples was conducted using LC‐MS/MS in NeuroVIS (Chungcheongnam‐do, Republic of Korea). **Table** **2** presents the analytical conditions of the LC‐MS/MS instrument. To separate GABA and glutamate in the LC‐MS/MS system composed of ExionLC Series UHPLC, AB SCIEX Triple Quadrupole 6500+, and ESI (SCIEX, Framingham), an ACQUITY UPLC HSS T3 column (2.1 × 100 mm, 1.8 µm, Waters, Milford, MA, USA) at 50 °C was used. The mobile phase consisted of 1) water containing 0.1% FA and 5 mm ammonium formate and 2) a 1:1 mixture of methanol and acetonitrile containing 5 mm ammonium formate. The flow rate was adjusted at 0.3 mL min^−1^, and a 7 µL per sample was injected in LC‐MS/MS. Multiple reaction monitoring (MRM) was performed in the positive mode for each NTs. The experiment was conducted at an ion transfer temperature of 500 °C, and a positive ion spray voltage of 5000 V.

**Table 2:** MRM transitions, retention times, and other conditions of GABA and glutamate.

Analyte	Q1 Mass (*m/z*)	Q3 Mass (*m/z*)	CE (V)	CXP (V)	Retention time (min)	Ionization polarity
GABA	104	87	15	10	0.97	Positive
Glutamate	148	84	25	10	0.98	Positive

### Statistical Analyses

All experimental data were analyzed using statistics software (IBM, SPSS Statistics 27). In order to determine whether the data from each group follows a normal distribution, the Shapiro‐Wilk test^[^ ^49^ ^]^ was performed. Once the normality was confirmed, the statistical significance of the difference between a sonicated group and the PTZ‐control group was assessed by *t*‐test. For the *t*‐test, a paired *t*‐test was employed in case the data were compared by time within the same group. If the distribution of the data departed significantly from the normality, a non‐parametric Mann–Whitney U test was performed to compare the significance of the difference. The type of test used for the statistical analysis was presented along with the p‐values throughout the paper. The purpose of experiments in this paper was to find either upregulating or downregulating effects for each stimulation sequence independently by comparing the effect with the control group. The neuromodulation effect for each stimulation sequence was only observed from a rat group of PTZ‐control that did not receive other stimulation sequences. Therefore, two‐sided Mann–Whitney U test was employed without considering multiple comparison corrections. *p* <0.05 was considered statistically significant and was marked as ** *p* <0.10 was considered trending toward statistical significance and was marked as *p > 0.10 was considered non‐significant and was marked as NS throughout the paper. Although *p* < 0.05 was the typical standard significance threshold in the general scientific community, the differences *p* <0.10 were also provisioned on the figures as providing the information may still identify potential trends – especially considering the small sample sizes – and suggest a potentially meaningful relationship that warrants further study.^[^ ^50^ ^]^ Effects with significance of *p* <0.10 were termed as “mild” throughout the paper. The sample sizes were determined by using the resource equation method.^[^ ^51^, ^52^ ^]^

## Conflict of Interest

The authors declare no conflict of interest.

## Supporting information

Supporting Information

Supplemental Video 1

Supplemental Video 2

Supplemental Video 3

Supplemental Video 4

Supplemental Video 5

Supplemental Video 6

Supplemental Video 7

## Data Availability

The data that support the findings of this study are available from the corresponding author upon reasonable request.
