# Supplementary material for: Bidirectional Neuronal Control of Epileptiform Activity by Repetitive Transcranial Focused Ultrasound Stimulations
Source: Adv Sci (Weinh). 2023 Nov 23;11(2):2302404. doi: 10.1002/advs.202302404 (PMC10787102; doi:10.1002/advs.202302404)
Supplement: Supplementary file 1 — Supporting Information [file ADVS-11-2302404-s001.pdf]

ADVANCED SCIENCE
Open Access

Supporting Information

for *Adv. Sci.*, DOI 10.1002/advs.202302404

Bidirectional Neuronal Control of Epileptiform Activity by Repetitive Transcranial Focused Ultrasound Stimulations

Taewon Choi, Minseok Koo, Jaesoon Joo, Taekyung Kim, Young-Min Shon and Jinhyoung Park**

ADVANCED SCIENCE
Open Access

Supplementary Materials for

Bidirectional Control of Epileptiform Activity by Repetitive Transcranial Focused Ultrasound Stimulations

T. Choi†, M. Koo†, J. Joo, T. Kim, Y. Shon, J. Park

Corresponding author: Jinhyoung Park, PhD
E-mail: jin.park@skku.edu

This PDF file includes:

Supplementary Figures 1 to 4

Supplementary Table 1

Supplementary Videos 1 to 7

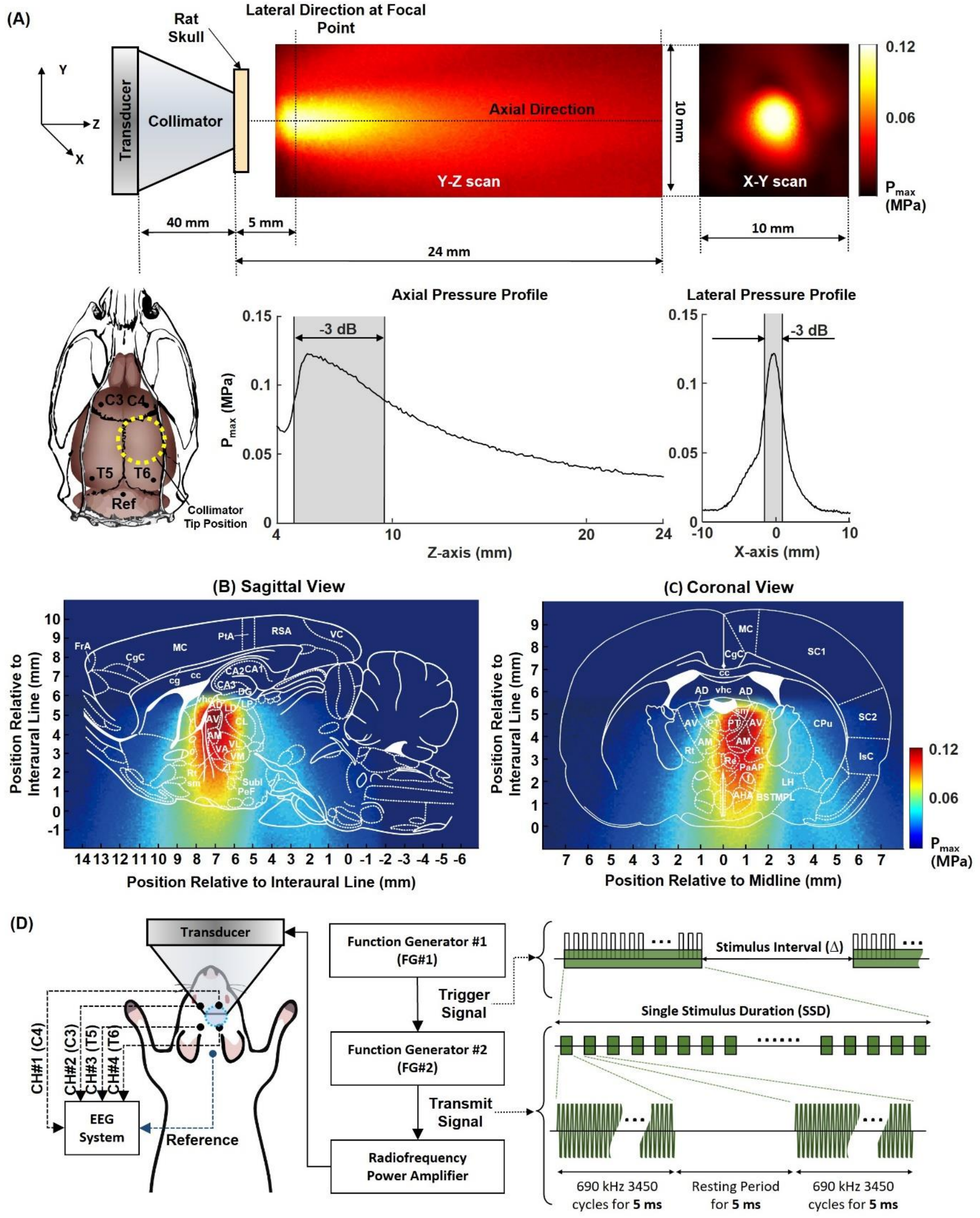

S. Fig. 1 Ultrasound transducer set up for the stimulation. A. Acoustic beam profile of the ultrasound transducer and the transducer tip and electrode positions in reference to the rat brain and skull. **B, C.** Sagittal and coronal views of the ultrasound beam superimposed to the Paxinos rat brain atlas [36]. **D.** Overall experimental system for ultrasound stimulations with

simultaneous four-channel EEG recording and signal shape generated from each function generator to obtain the stimulation pulse sequence.

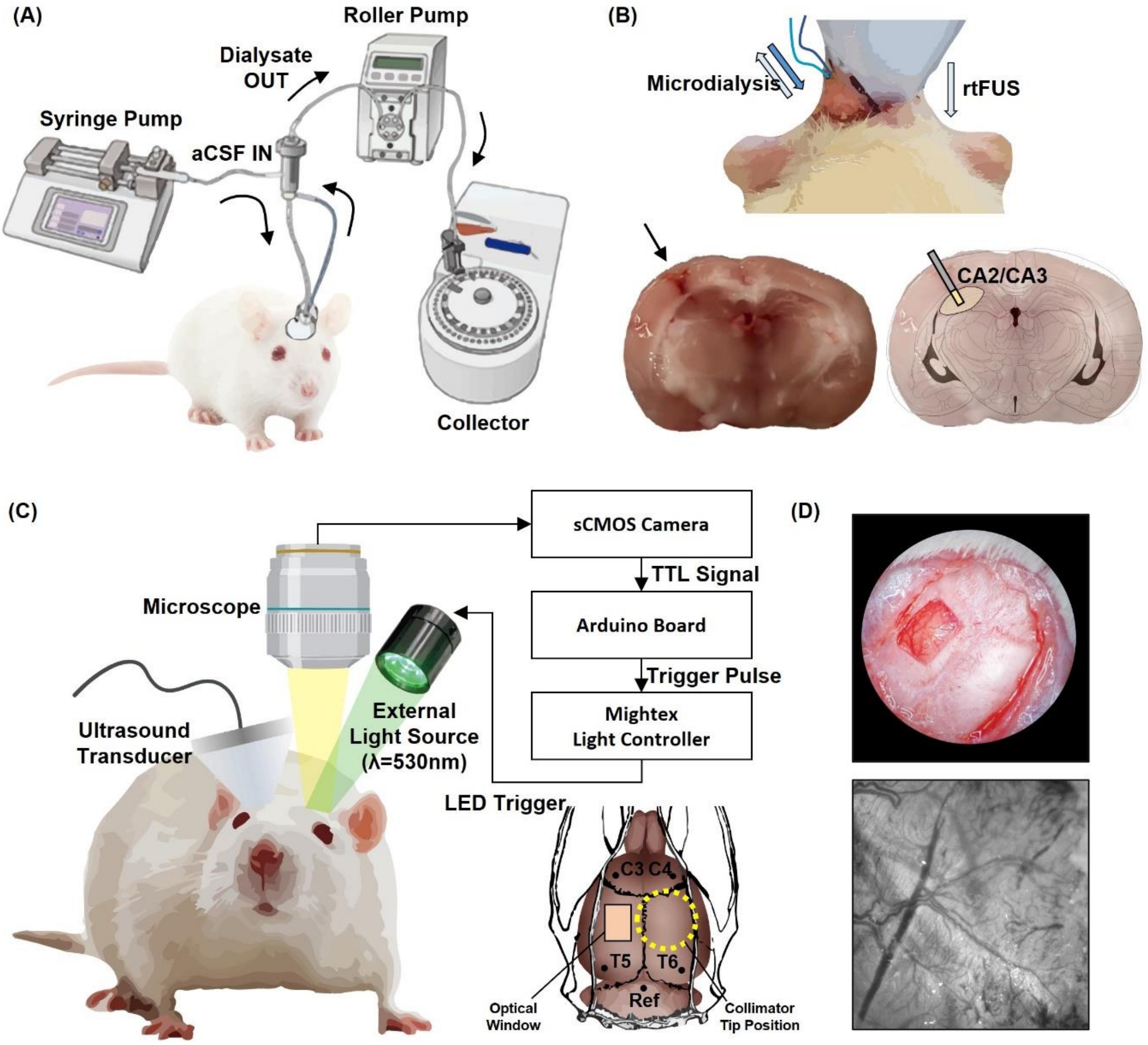

S. Fig. 2 Experimental description of the microdialysis for proteomic analysis of neurotransmitters and optical measurement of cerebral blood volume changes. A. Experimental set up for the microdialysis to allow aCSF flow through syringe pump, brain parenchyma, roller pump and the collector. **B.** Experimental set up of the ultrasound transducer and the microdialysis probe. A 1-mm membrane probe was inserted to target the CA2 and CA3 region with a tilted angle of 26 degrees to allow the positioning of the ultrasound transducer. **C.** Recording system for the cerebral blood volume measurement. Green (530 nm) light was applied to the cranial window to measure the changes in the local total blood volume. **D.** Optical window was made on the left hemisphere side to allow the ultrasound transducer to position at the right ATN region.

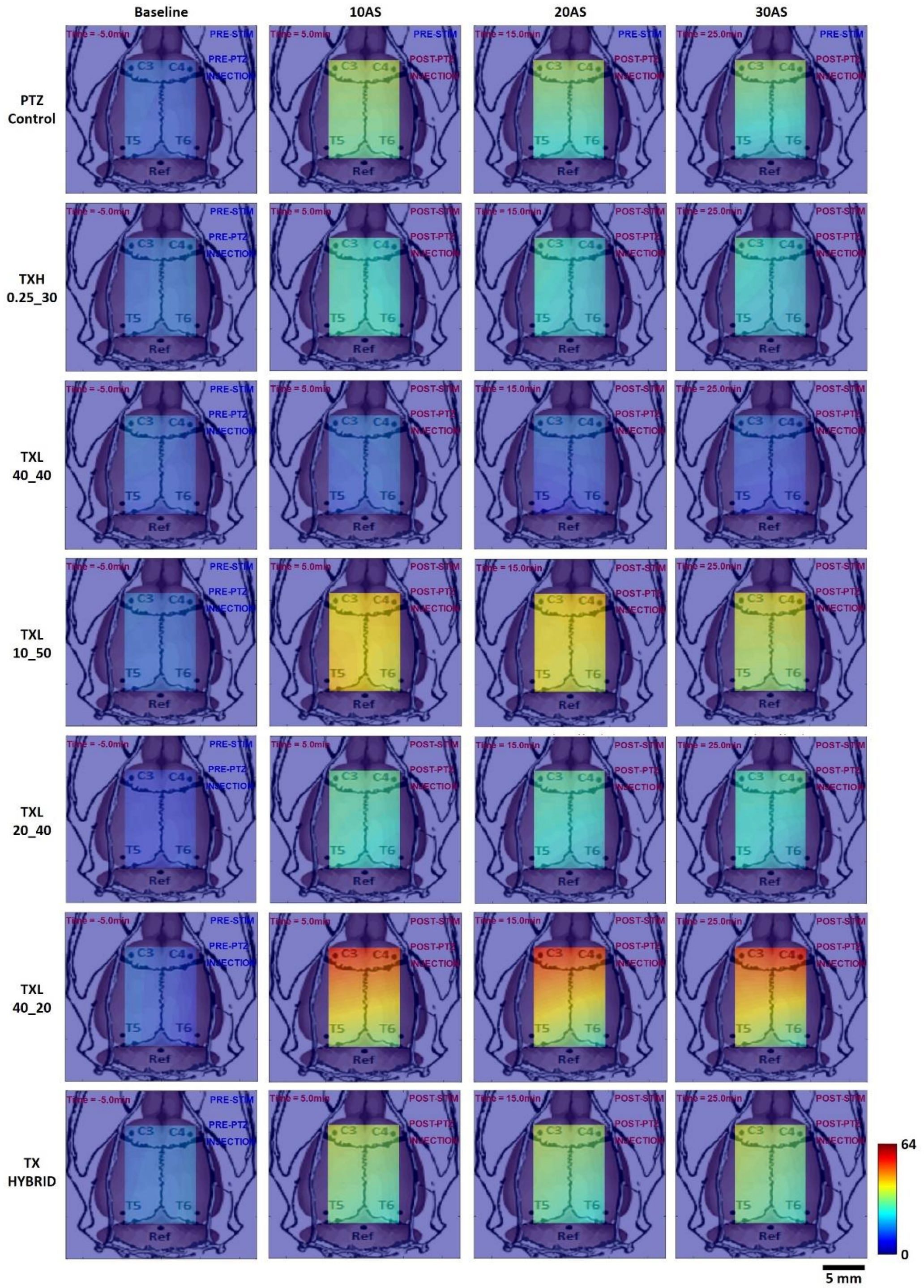

S. Fig. 3 Two-dimensional topographical maps representing the number of epileptic spikes by time measured at electrodes in C3, C4, T5, and T6. The color map images were acquired by interpolating the number of epileptic spikes acquired with EEG signals from the four electrodes. The measured numbers were placed at the four corners of the topological window, and the two-dimensional information was calculated by linear interpolation. The representative images at baseline and 10 (10AS), 20 (20AS), and 30 (30AS) min after stimulus are presented. Animated videos could be found in S. Vids. 1–7.

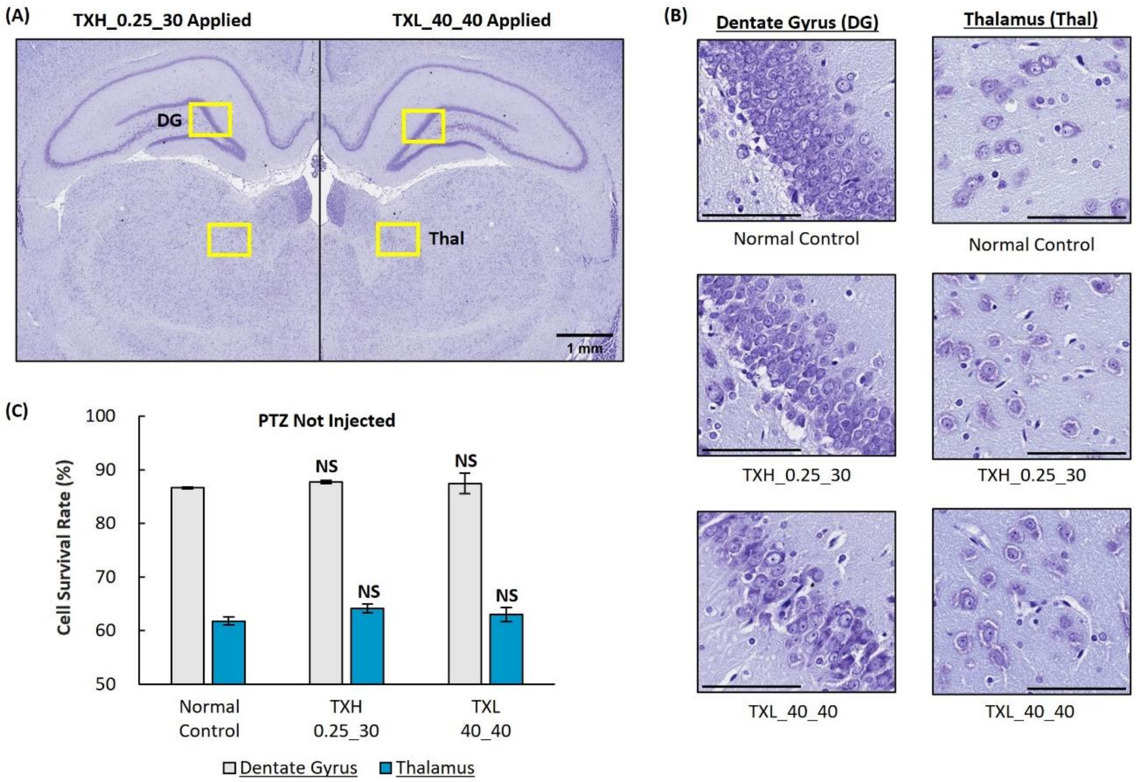

S. Fig. 4 Cresyl Violet (Nissl) staining analysis results. A. Representative images of Nissl-stained brain slice and the regions for analysis. **B.** Representative images of Nissl-stained brain slice images at the hippocampus and thalamic region. **C.** The Cresyl Violet Staining analysis demonstrated no damages in the rat brain after both TXH_0.25_30 and TXL_40_40 ultrasonic stimulation (n = 3). The scale bar denotes 100 µm for all magnified images. Data presented as mean ± SEM, p-values calculated via two-tailed Mann-Whitney U test, **NS** : $p > 0.10$.

S. Table 1. Abbreviation List

Abbreviation	Full Name	Abbreviation	Full Name
**AED**	antiepileptic drug	**ML**	laterally from the bregma
**AP**	anteriorly (+) or posteriorly (-) from the bregma	**PRF**	pulse repetition frequency
**AS**	minutes after stimulations	**PTZ**	pentylenetetrazol
**ATN**	anterior thalamic nuclei	**rtFUS**	repetitive transcranial focused ultrasound stimulation
**DBS**	deep brain stimulation	**SBRI**	Samsung Biomedical Research Institute
**DV**	dorsoventral distance from the horizontal plane passing through the bregma and lambda on the surface of the skull	**SD rat**	Sprague-Dawley rat
**EEG**	electroencephalography	**tACS**	transcranial alternating current stimulation
**FG**	function generator	**tDCS**	transcranial direct current stimulation
**GABA**	gamma-aminobutyric acid	**tFUS**	transcranial focused ultrasound stimulation
**GAD65**	glutamic acid decarboxylase 65-kilodalton isoform	**TLE**	temporal lobe epilepsy
**GFAP**	glial fibrillary acidic protein	**TMS**	transcranial magnetic stimulation
**IACUC**	Institutional Animal Care and Use Committees	**TX**	ultrasound pulse transmit sequence
**Iba1**	ionized calcium binding adaptor molecule 1	**TXL**	ultrasound pulse transmit sequence that applies lower acoustic peak negative maximum pressure (0.25 MPa)
**IHC**	immunohistochemistry	**TXH**	ultrasound pulse transmit sequences that apply higher acoustic peak negative maximum pressure (1.0 MPa)
**ILAR**	Institute of Laboratory Animal Resources	**VNS**	vagus nerve stimulation

S. Vid. 1.

2D topographical map representing the number of epileptic spikes ratio by time in PTZ-control.

https://drive.google.com/file/d/1KPbIDVXFsXd-WbUvzM0sobk2Aqnadtv8/view?usp=share_link

S. Vid. 2.

2D topographical map representing the number of epileptic spikes ratio by time in TXH_0.25_30.

https://drive.google.com/file/d/1DlTAauB85U_nfXVUGiTY4zv2VMoS72H0/view?usp=share_link

S. Vid. 3.

2D topographical map representing the number of epileptic spikes ratio by time in TXL_40_40.

https://drive.google.com/file/d/1JosXhozui0KUswy_N2UDRqZB05hKFALz/view?usp=share_link

S. Vid. 4.

2D topographical map representing the number of epileptic spikes ratio by time in TX_10_50.

https://drive.google.com/file/d/1Z6X3aiaK_o2Rrz14QDR0P5fn-oJ8izbZ/view?usp=share_link

S. Vid. 5.

2D topographical map representing the number of epileptic spikes ratio by time in TXL_20_40.

https://drive.google.com/file/d/1hYAgjBoCCMrZmJUYuDMVLoCbjAjBkmsB/view?usp=share_link

S. Vid. 6.

2D topographical map representing the number of epileptic spikes ratio by time in TXL_40_20.

https://drive.google.com/file/d/1MYI2SgAlxL9nJUx0ZZpE9VUPIfVBDixx/view?usp=share_link

S. Vid. 7.

2D topographical map representing the number of epileptic spikes ratio by time in TX_HYBRID.

https://drive.google.com/file/d/1lZInWVtR9k1vmNO31jR3EpO2phkcDTyG/view?usp=share_link
